# Supplementary material for: Hot spot prediction in protein-protein interactions by an ensemble system
Source: BMC Syst Biol. 2018 Dec 31;12(Suppl 9):132. doi: 10.1186/s12918-018-0665-8 (PMC6311905; doi:10.1186/s12918-018-0665-8)
Supplement: Supplementary file 2 — All relevant MATLAB code. A simple Matlab implement of our predictor. (ZIP 19,742 kb) [file 12918_2018_665_MOESM2_ESM.zip › Additional file 2/Read ASA/ASA website/dbMPIKT/4P5TD.html]

 NetSurfP - Protein Surface Accessibility and Secondary Structure Predictions

  

|  |  |  |
| --- | --- | --- |
|  |  | NetSurfP - Protein Surface Accessibility and Secondary Structure Predictions  Technical University of Denmark |

  


---

```
# For publication of results, please cite:
# A generic method for assignment of reliability scores applied to solvent accessibility predictions.
# Bent Petersen, Thomas Nordahl Petersen, Pernille Andersen, Morten Nielsen and Claus Lundegaard
# BMC Structural Biology 2009, 9:51 doi:10.1186/1472-6807-9-51
#
# Column 1: Class assignment - B for buried or E for Exposed - Threshold: 25% exposure, but not based on RSA
# Column 2: Amino acid
# Column 3: Sequence name
# Column 4: Amino acid number
# Column 5: Relative Surface Accessibility - RSA
# Column 6: Absolute Surface Accessibility
# Column 7: Z-fit score for RSA prediction
# Column 8: Probability for Alpha-Helix
# Column 9: Probability for Beta-strand
# Column 10: Probability for Coil
E G  4P5TD                  1    0.837  65.911  -2.350   0.003   0.003   0.994
E G  4P5TD                  2    0.682  53.673  -2.910   0.005   0.015   0.979
E S  4P5TD                  3    0.395  46.306  -1.811   0.018   0.047   0.935
E E  4P5TD                  4    0.589 102.898  -1.279   0.053   0.043   0.903
E R  4P5TD                  5    0.344  78.776  -1.531   0.052   0.084   0.864
B H  4P5TD                  6    0.225  40.946   0.296   0.069   0.386   0.545
B F  4P5TD                  7    0.106  21.194   0.696   0.018   0.846   0.136
B V  4P5TD                  8    0.124  18.982   0.467   0.011   0.918   0.071
B Y  4P5TD                  9    0.065  13.933   1.147   0.001   0.959   0.040
B Q  4P5TD                 10    0.068  12.073   0.704   0.001   0.959   0.040
B F  4P5TD                 11    0.129  25.850   0.478   0.001   0.959   0.040
B M  4P5TD                 12    0.095  18.929   0.027   0.001   0.959   0.040
B G  4P5TD                 13    0.060   4.714  -0.086   0.001   0.900   0.099
B E  4P5TD                 14    0.036   6.307  -0.949   0.001   0.900   0.099
B C  4P5TD                 15    0.020   2.766   0.925   0.001   0.959   0.040
B Y  4P5TD                 16    0.141  30.068  -0.120   0.001   0.959   0.040
B F  4P5TD                 17    0.055  11.139   0.424   0.001   0.900   0.099
B T  4P5TD                 18    0.224  30.999   0.285   0.004   0.514   0.481
E N  4P5TD                 19    0.668  97.737  -0.983   0.004   0.085   0.910
E G  4P5TD                 20    0.743  58.513  -2.377   0.005   0.015   0.979
E T  4P5TD                 21    0.421  58.448  -1.775   0.005   0.045   0.951
E Q  4P5TD                 22    0.452  80.656  -1.200   0.005   0.262   0.733
E R  4P5TD                 23    0.452 103.554  -0.154   0.004   0.514   0.481
B I  4P5TD                 24    0.186  34.484  -0.304   0.004   0.616   0.381
E R  4P5TD                 25    0.324  74.173  -0.164   0.002   0.816   0.182
B Y  4P5TD                 26    0.159  33.893  -0.563   0.001   0.900   0.099
B V  4P5TD                 27    0.133  20.411   0.170   0.001   0.959   0.040
B T  4P5TD                 28    0.169  23.482  -0.009   0.001   0.959   0.040
B R  4P5TD                 29    0.146  33.503   0.428   0.001   0.959   0.040
B Y  4P5TD                 30    0.108  23.101   0.173   0.001   0.959   0.040
B I  4P5TD                 31    0.124  22.866  -0.870   0.001   0.900   0.099
B Y  4P5TD                 32    0.193  41.265  -0.075   0.004   0.616   0.381
E N  4P5TD                 33    0.467  68.427  -0.268   0.004   0.138   0.858
E R  4P5TD                 34    0.382  87.455  -1.070   0.018   0.088   0.893
E E  4P5TD                 35    0.437  76.414  -0.189   0.004   0.514   0.481
B E  4P5TD                 36    0.229  39.971  -0.250   0.002   0.816   0.182
B Y  4P5TD                 37    0.124  26.456   0.814   0.011   0.918   0.071
B V  4P5TD                 38    0.064   9.806   0.151   0.001   0.959   0.040
B R  4P5TD                 39    0.185  42.479   0.213   0.001   0.959   0.040
B Y  4P5TD                 40    0.055  11.732   0.787   0.001   0.900   0.099
E D  4P5TD                 41    0.244  35.160   0.049   0.002   0.816   0.182
B S  4P5TD                 42    0.215  25.233  -0.862   0.022   0.552   0.426
E D  4P5TD                 43    0.556  80.062  -1.159   0.021   0.279   0.699
E V  4P5TD                 44    0.336  51.705  -0.796   0.052   0.084   0.864
E G  4P5TD                 45    0.412  32.424  -1.197   0.052   0.084   0.864
E E  4P5TD                 46    0.403  70.422  -1.338   0.056   0.142   0.802
B H  4P5TD                 47    0.206  37.490  -0.870   0.064   0.216   0.721
E R  4P5TD                 48    0.377  86.402  -0.747   0.064   0.216   0.721
B A  4P5TD                 49    0.211  23.263  -1.907   0.118   0.150   0.732
E V  4P5TD                 50    0.285  43.774  -1.109   0.191   0.086   0.723
E T  4P5TD                 51    0.308  42.664  -0.485   0.354   0.048   0.598
E E  4P5TD                 52    0.346  60.446  -1.374   0.455   0.046   0.498
B L  4P5TD                 53    0.278  50.847  -0.820   0.522   0.016   0.462
B G  4P5TD                 54    0.256  20.187  -2.313   0.561   0.047   0.393
E R  4P5TD                 55    0.336  76.898  -0.629   0.561   0.047   0.393
E P  4P5TD                 56    0.364  51.680  -0.897   0.561   0.047   0.393
E D  4P5TD                 57    0.296  42.668  -0.831   0.660   0.049   0.291
E A  4P5TD                 58    0.296  32.619  -0.866   0.660   0.049   0.291
E E  4P5TD                 59    0.492  85.970  -0.996   0.622   0.015   0.363
E Y  4P5TD                 60    0.366  78.150  -0.629   0.660   0.049   0.291
E W  4P5TD                 61    0.301  72.390  -0.576   0.522   0.016   0.462
E N  4P5TD                 62    0.424  62.000  -1.018   0.430   0.016   0.555
E S  4P5TD                 63    0.367  42.989  -0.849   0.455   0.046   0.498
E Q  4P5TD                 64    0.342  60.992  -0.605   0.386   0.097   0.517
E P  4P5TD                 65    0.322  45.692  -0.987   0.307   0.165   0.527
E E  4P5TD                 66    0.360  62.875  -0.873   0.428   0.171   0.402
B I  4P5TD                 67    0.272  50.376  -0.347   0.538   0.173   0.289
B L  4P5TD                 68    0.255  46.764  -0.683   0.502   0.102   0.396
E E  4P5TD                 69    0.338  59.136  -0.650   0.561   0.047   0.393
E R  4P5TD                 70    0.360  82.325  -1.015   0.502   0.102   0.396
E T  4P5TD                 71    0.355  49.238  -0.547   0.428   0.171   0.402
E R  4P5TD                 72    0.307  70.303  -0.183   0.428   0.171   0.402
E A  4P5TD                 73    0.369  40.708  -0.334   0.307   0.165   0.527
E E  4P5TD                 74    0.369  64.464  -0.898   0.199   0.152   0.649
E L  4P5TD                 75    0.372  68.113  -0.830   0.191   0.086   0.723
E D  4P5TD                 76    0.393  56.675  -0.791   0.191   0.086   0.723
E T  4P5TD                 77    0.474  65.688  -0.910   0.191   0.086   0.723
E V  4P5TD                 78    0.338  51.874  -0.699   0.191   0.086   0.723
E C  4P5TD                 79    0.357  50.123  -0.766   0.191   0.086   0.723
E R  4P5TD                 80    0.383  87.661  -0.924   0.191   0.086   0.723
E H  4P5TD                 81    0.374  68.122  -0.958   0.191   0.086   0.723
E N  4P5TD                 82    0.325  47.595  -0.873   0.191   0.086   0.723
E Y  4P5TD                 83    0.342  73.064  -0.854   0.191   0.086   0.723
E E  4P5TD                 84    0.370  64.639  -1.027   0.191   0.086   0.723
E G  4P5TD                 85    0.382  30.032  -0.982   0.191   0.086   0.723
E P  4P5TD                 86    0.314  44.585  -1.026   0.191   0.086   0.723
E E  4P5TD                 87    0.380  66.299  -0.921   0.191   0.086   0.723
E T  4P5TD                 88    0.380  52.734  -1.093   0.191   0.086   0.723
E H  4P5TD                 89    0.373  67.776  -1.204   0.191   0.086   0.723
E T  4P5TD                 90    0.328  45.480  -0.767   0.184   0.043   0.773
E S  4P5TD                 91    0.547  64.097  -0.655   0.278   0.093   0.628
E L  4P5TD                 92    0.458  83.787  -0.881   0.191   0.086   0.723
E R  4P5TD                 93    0.381  87.226  -0.969   0.191   0.086   0.723
E R  4P5TD                 94    0.402  92.150  -1.304   0.113   0.087   0.800
E L  4P5TD                 95    0.590 108.121  -1.004   0.052   0.084   0.864
E E  4P5TD                 96    0.468  81.847  -0.612   0.052   0.084   0.864
E Q  4P5TD                 97    0.498  88.854  -0.460   0.018   0.088   0.893
E P  4P5TD                 98    0.414  58.789  -1.315   0.018   0.088   0.893
E N  4P5TD                 99    0.437  63.977  -0.461   0.021   0.279   0.699
B V  4P5TD                100    0.270  41.438  -1.460   0.004   0.514   0.481
E V  4P5TD                101    0.335  51.459  -0.798   0.003   0.718   0.279
B I  4P5TD                102    0.174  32.227  -2.123   0.002   0.816   0.182
B S  4P5TD                103    0.230  26.944  -1.502   0.002   0.816   0.182
B L  4P5TD                104    0.257  47.020  -0.952   0.002   0.816   0.182
E S  4P5TD                105    0.370  43.317  -0.969   0.023   0.655   0.322
E R  4P5TD                106    0.451 103.233  -1.143   0.023   0.655   0.322
E T  4P5TD                107    0.503  69.766  -1.154   0.004   0.514   0.481
E E  4P5TD                108    0.393  68.675  -1.954   0.021   0.279   0.699
E A  4P5TD                109    0.538  59.277  -1.131   0.018   0.088   0.893
E L  4P5TD                110    0.490  89.646  -1.919   0.018   0.019   0.964
E N  4P5TD                111    0.741 108.453  -1.131   0.005   0.015   0.979
E H  4P5TD                112    0.571 103.865  -1.509   0.005   0.045   0.951
E H  4P5TD                113    0.446  81.182  -0.065   0.005   0.262   0.733
B N  4P5TD                114    0.134  19.559  -0.365   0.003   0.718   0.279
E T  4P5TD                115    0.249  34.550   1.026   0.002   0.816   0.182
B L  4P5TD                116    0.023   4.266   0.857   0.001   0.900   0.099
B V  4P5TD                117    0.078  11.942   0.559   0.001   0.959   0.040
B C  4P5TD                118    0.017   2.387   1.065   0.001   0.959   0.040
B S  4P5TD                119    0.135  15.810   0.608   0.001   0.959   0.040
B V  4P5TD                120    0.026   4.073  -0.498   0.001   0.900   0.099
B T  4P5TD                121    0.218  30.250   0.092   0.003   0.718   0.279
E D  4P5TD                122    0.480  69.154  -0.789   0.005   0.336   0.660
B F  4P5TD                123    0.176  35.363  -1.580   0.004   0.138   0.858
E Y  4P5TD                124    0.404  86.356  -0.405   0.004   0.138   0.858
B P  4P5TD                125    0.258  36.568  -0.668   0.005   0.045   0.951
E A  4P5TD                126    0.513  56.489  -0.930   0.004   0.138   0.858
E K  4P5TD                127    0.446  91.742  -0.015   0.004   0.616   0.381
B I  4P5TD                128    0.028   5.106   0.346   0.001   0.900   0.099
E K  4P5TD                129    0.281  57.843   1.300   0.001   0.959   0.040
B V  4P5TD                130    0.028   4.304   0.659   0.001   0.959   0.040
B R  4P5TD                131    0.221  50.586   1.710   0.001   0.959   0.040
B W  4P5TD                132    0.059  14.093   0.937   0.001   0.959   0.040
E F  4P5TD                133    0.269  53.948   0.998   0.001   0.959   0.040
B R  4P5TD                134    0.223  50.998   0.396   0.002   0.816   0.182
E N  4P5TD                135    0.563  82.467  -0.543   0.005   0.045   0.951
E G  4P5TD                136    0.675  53.115  -1.370   0.005   0.015   0.979
E Q  4P5TD                137    0.575 102.695   0.226   0.004   0.514   0.481
E E  4P5TD                138    0.625 109.170  -0.061   0.004   0.616   0.381
E E  4P5TD                139    0.226  39.500   0.670   0.022   0.359   0.619
E T  4P5TD                140    0.471  65.355  -0.332   0.020   0.205   0.775
E V  4P5TD                141    0.623  95.694  -0.457   0.019   0.141   0.840
E G  4P5TD                142    0.670  52.753  -1.585   0.020   0.205   0.775
E V  4P5TD                143    0.314  48.339  -0.562   0.004   0.420   0.576
E S  4P5TD                144    0.525  61.542  -0.896   0.004   0.616   0.381
E S  4P5TD                145    0.442  51.826  -1.073   0.004   0.514   0.481
E T  4P5TD                146    0.367  50.945  -1.915   0.022   0.359   0.619
E Q  4P5TD                147    0.503  89.818  -0.902   0.022   0.552   0.426
E L  4P5TD                148    0.355  64.946  -0.593   0.023   0.655   0.322
E I  4P5TD                149    0.324  59.977  -0.167   0.023   0.655   0.322
E R  4P5TD                150    0.396  90.776  -0.935   0.004   0.514   0.481
E N  4P5TD                151    0.555  81.223  -0.616   0.004   0.197   0.799
E G  4P5TD                152    0.665  52.335  -1.676   0.005   0.015   0.979
E D  4P5TD                153    0.718 103.536  -1.545   0.003   0.003   0.994
E W  4P5TD                154    0.330  79.293  -0.739   0.004   0.138   0.858
B T  4P5TD                155    0.176  24.370   0.249   0.003   0.718   0.279
B F  4P5TD                156    0.059  11.841   0.276   0.001   0.900   0.099
B Q  4P5TD                157    0.107  19.021   0.622   0.001   0.959   0.040
B V  4P5TD                158    0.064   9.760   0.008   0.001   0.959   0.040
B L  4P5TD                159    0.134  24.480   0.337   0.001   0.959   0.040
B V  4P5TD                160    0.043   6.548  -0.476   0.001   0.959   0.040
B M  4P5TD                161    0.148  29.655   0.600   0.001   0.959   0.040
B L  4P5TD                162    0.035   6.482  -0.246   0.001   0.959   0.040
E E  4P5TD                163    0.361  63.014   0.769   0.001   0.959   0.040
B M  4P5TD                164    0.051  10.145   1.006   0.002   0.816   0.182
E T  4P5TD                165    0.323  44.800   1.313   0.005   0.336   0.660
E P  4P5TD                166    0.390  55.298  -0.186   0.004   0.085   0.910
E R  4P5TD                167    0.713 163.185   0.575   0.005   0.015   0.979
E R  4P5TD                168    0.645 147.819   0.213   0.005   0.015   0.979
E G  4P5TD                169    0.636  50.069  -0.448   0.005   0.015   0.979
E E  4P5TD                170    0.507  88.520   0.194   0.004   0.197   0.799
E V  4P5TD                171    0.460  70.671   1.753   0.002   0.816   0.182
B Y  4P5TD                172    0.062  13.207   0.964   0.001   0.900   0.099
B T  4P5TD                173    0.236  32.761   1.233   0.001   0.959   0.040
B C  4P5TD                174    0.018   2.541   1.050   0.001   0.959   0.040
B H  4P5TD                175    0.227  41.237   1.659   0.001   0.959   0.040
B V  4P5TD                176    0.037   5.625   0.247   0.001   0.959   0.040
E E  4P5TD                177    0.357  62.438   0.781   0.001   0.959   0.040
B H  4P5TD                178    0.087  15.843  -0.365   0.002   0.816   0.182
E P  4P5TD                179    0.512  72.695  -0.027   0.004   0.197   0.799
E S  4P5TD                180    0.447  52.377  -0.973   0.004   0.085   0.910
B L  4P5TD                181    0.203  37.133   0.153   0.004   0.085   0.910
E K  4P5TD                182    0.668 137.510   0.342   0.018   0.047   0.935
E S  4P5TD                183    0.529  61.987   0.482   0.018   0.047   0.935
E P  4P5TD                184    0.519  73.660  -0.149   0.019   0.141   0.840
B I  4P5TD                185    0.193  35.742   0.608   0.004   0.616   0.381
E T  4P5TD                186    0.410  56.895   0.553   0.002   0.816   0.182
E V  4P5TD                187    0.332  51.090   1.771   0.011   0.918   0.071
E E  4P5TD                188    0.497  86.896   1.201   0.011   0.918   0.071
B W  4P5TD                189    0.157  37.638   1.048   0.021   0.756   0.223
E R  4P5TD                190    0.571 130.759   0.596   0.004   0.616   0.381
E A  4P5TD                191    0.724  79.741  -1.316   0.003   0.003   0.994

Explain the output.  Go back.
```

---
